# 6q24-Related Transient Neonatal Diabetes Mellitus Presenting With Severe Diabetic Ketoacidosis and Multiorgan Failure

**DOI:** 10.1210/jcemcr/luaf145

**Published:** 2025-09-18

**Authors:** Bassem M Shoucri, Apisadaporn Thambundit, Dennis J Chia

**Affiliations:** Department of Pediatrics, Division of Pediatric Endocrinology, UCLA David Geffen School of Medicine, Los Angeles, CA 90095, USA; Department of Pediatrics, Division of Pediatric Endocrinology, UCLA David Geffen School of Medicine, Los Angeles, CA 90095, USA; Department of Pediatrics, Division of Pediatric Endocrinology, UCLA David Geffen School of Medicine, Los Angeles, CA 90095, USA

**Keywords:** neonatal diabetes, neonatal hyperglycemia, uniparental disomy, diabetic ketoacidosis, neonatal thalamic hemorrhage

## Abstract

Neonatal diabetes is commonly defined by presentation in the first 6 months of life, with subsets of permanent and transient neonatal diabetes mellitus (TNDM) distinguished by spontaneous resolution of hyperglycemia in the latter. Infants with TNDM frequently require hospitalization for rehydration but characteristically do not present with a severe clinical course with ketoacidosis. Here we present a 19-day-old infant with a history of intrauterine growth restriction, small for gestational age, and normal blood sugars in the first 25 hours of life who presented with respiratory distress that rapidly progressed to shock and multiorgan failure. Diagnostic studies on presentation revealed severe hyperglycemia, metabolic acidosis, transaminitis, acute kidney injury, hyperammonemia, and thalamic hyperintensities that collectively raised suspicion for a metabolic/mitochondrial disorder. The patient's clinical course and genetic studies, however, converged on a diagnosis of TNDM due to uniparental disomy of chromosome 6. Diabetes has resolved, but the patient has endured permanent motor deficits associated with thalamic hemorrhage at presentation. This case highlights that TNDM can escape detection by blood glucose measurements in the first days of life and indeed present in severe diabetic ketoacidosis, which can further progress to shock and multiorgan failure that may mimic a metabolic/mitochondrial disorder.

## Introduction

Diabetes mellitus presenting within the first 6 months of life, classified as neonatal diabetes mellitus (NDM), most often results from a heterogenous group of monogenic and imprinting abnormalities [1-3 ]. As highlighted by the nomenclature, subsets of transient and permanent NDM are distinguished by their natural history, where TNDM evolves with resolution of hyperglycemia off exogenous insulin before 18 months, only to typically recur with permanent diabetes in later life [3]. Approximately 70% of TNDM cases are attributed to aberrations affecting the imprinted 6q24 locus [4], leading to overexpression of pleomorphic adenoma gene-like 1 (*PLAGL1*, formerly *ZAC*) and hyatidiform mole associated and imprinted (*HYMAI*) genes [5-7]. 6q24-related TNDM characteristically presents earlier than permanent NDM and with the absence of ketoacidosis [2, 3, 8, 9]. Here, we describe an infant who presented at 19 days of life with severe diabetic ketoacidosis (DKA) and multiorgan failure, where the natural history and genetic studies revealed the underlying mechanism as TNDM from paternal isodisomy of chromosome 6 (UPD6pat).

## Case Presentation

Our patient was a baby boy delivered at term vaginally without complication, following a pregnancy notable for intrauterine growth restriction and maternal hypothyroidism well controlled on levothyroxine. A prior pregnancy was similarly complicated by intrauterine growth restriction but otherwise completed with an unremarkable neonatal course. Birth parameters for the infant met criteria for small for gestational age (SGA) with weight 1990g (0.17 percentile on the Fenton growth curve), length 44 cm (0.84 percentile), and head circumference 31 cm (1.61 percentile). He was admitted to the neonatal intensive care unit per protocol for birth weight less than 2 kg. The infant briefly received oxygen support with high-flow nasal cannula but was readily weaned to room air by 12 hours of life. Capillary blood glucose levels were monitored regularly to identify hypoglycemia per protocol for SGA infants, with 10 readings within the first 25 hours ranging from 55 to 99 mg/dL (SI: 3.1-5.5 mmol/L). Following an otherwise unremarkable neonatal course, he was discharged home on day of life 2. The patient was followed closely by his outpatient pediatrician with follow-up visits on days of life 3, 6, and 14, where weight at the last visit had increased 15% from birth weight.

On day of life 19, the patient was brought to the emergency department for symptoms of respiratory distress. A chest x-ray revealed bilateral peribronchial thickening and interstitial opacities, and the patient was admitted to the pediatric floor for a presumed viral bronchiolitis. Within hours, however, the patient required increasing respiratory support, necessitating transfer to the pediatric intensive care unit for impending respiratory failure with rapid deterioration to shock.

## Diagnostic Assessment

Additional laboratory studies were obtained (Table 1), demonstrating impressive hyperglycemia, severe anion gap metabolic acidosis with elevated lactate and ketones (the latter inferred from urine organic acids), transaminitis, hyperammonemia, and acute kidney injury. Cerebrospinal fluid analysis revealed leukocytosis, elevated protein, elevated glucose, and elevated lactate. Coagulation studies demonstrated an elevated prothrombin time and international normalized ratio as well as low fibrinogen levels requiring fresh frozen plasma. The full infectious workup ultimately returned negative, including blood, urine, respiratory, and cerebrospinal fluid cultures, a meningoencephalitis panel, and a respiratory pathogen panel.

**Table 1.** Laboratory findings on admission to pediatric intensive care unit

		Reference range
Hematology		
White blood cell count	17 550 cells/µL	5000-20 000 cells/µL
Hemoglobin	11.2 g/dL (SI: 112 g/L)	12.5-20.5 g/dL (SI: 125-205 g/L)
Hematocrit	39.3%	39-66%
Platelet count	660 platelets/µL	143-398 platelets/µL
Coagulation		
Prothrombin time	21.7 seconds	13.5-16.4 seconds
International normalized ratio	2.0	N/A
Activated partial thromboplastin time	41.9 seconds	29.5-42.2 seconds
Fibrinogen	142 mg/dL (SI: 1.42 g/L)	283-401 mg/dL (SI: 2.83-4.01 g/L)
Antithrombin III activity	65.0%	60.0-89.0%
Protein C, functional	38.0%	24.0-51.0%
Chemistry		
Sodium	145 mmol/L	135-146 mmol/L
Potassium	5.8 mmol/L	3.6-5.3 mmol/L
Chloride	107 mmol/L	96-106 mmol/L
Bicarbonate	10 mmol/L	20-30 mmol/L
Blood urea nitrogen	36 mg/dL (SI: 12.9 mmol/L)	5-25 mg/dL (SI: 1.8-8.9 mmol/L)
Creatinine	1.14 mg/dL (SI: 101 µmol/L)	0.6-1.3 mg/dL (SI: 53-115 µmol/L)
Glucose	1260 mg/dL (SI: 70 mmol/L)	65-99 mg/dL (SI: 3.6-5.5 mmol/L)
Calcium	7.6 mg/dL (SI: 1.9 mmol/L)	8.6-11.0 mg/dL (SI: 2.1-2.7 mmol/L)
Aspartate aminotransferase	518 U/L	<64 U/L
Alanine aminotransferase	102 U/L	<26 U/L
Alkaline phosphatase	703 U/L	122-469 U/L
Total bilirubin	0.5 mg/dL (SI: 8.55 µmol/L)	<0.7 (SI: < 12 µmol/L)
Direct bilirubin	0.3 mg/dL (SI: 5.1 µmol/L)	≤0.3 (SI: < 5.1 µmol/L)
Albumin	2.3 g/L	3.9-5.0 g/L
Ammonia	139 µg/dL (SI: 81.6 µmol/L)	30-90 µg/dL (SI: 17.6-52.9 µmol/L)
Lactate	86 mg/dL (SI: 9.6 mmol/L)	5-25 mg/dL (SI: 0.6-2.8 mmol/L)
Insulin	<1 µU/mL (SI: <7 pmol/L)	3-25 µU/mL (SI: 21-174 pmol/L)
C-peptide	0.6 ng/mL (SI: 0.2 nmol/L)	1.1-4.3 ng/mL (SI: 0.4-1.4 nmol/L)
Urine		
pH	6.0	5.0-8.0
Specific gravity	1.024	1.005-1.030
Blood	2+	negative
Glucose	3+	negative
Ketones	1+	negative
Protein	1+	negative
Nitrites	negative	negative
Leukocytes	negative	negative
3-hydroxybutyric acid	5385 mmol/mol Cr	≤15 mmol/mol Cr
Acetoacetic acid	1275 mmol/mol Cr	≤10 mmol/mol Cr
Venous blood gas		
pH	6.92	7.37-7.41
Carbon dioxide	23 mmHg	38-42 mmHg
Bicarbonate	4.7 mmol/L	22-26 mmol/L
Base excess	−26 mmol/L	−2 − + 2 mmol/L
Cerebrospinal fluid		
Red blood cell count	103 cells/mm^3^	0-10 cells/mm^3^
White blood cell count	216 cells/mm^3^	0-20 cells/mm^3^
Segmented neutrophils	83%	N/A
Glucose	859 mg/dL (SI: 47.7 mmol/L)	43-73 mg/dL (SI: 2.4-4.1 mmol/L)
Protein	1865 mg/dL (SI: 186.5 mg/L)	14-45 mg/dL (SI: 1.4-4.5 mg/L)
Lactate	99 mg/dL (SI: 11.1 mmol/L)	10-25 mg/dL (SI: 1.1-2.8 mmol/L)

**Figure 1.**
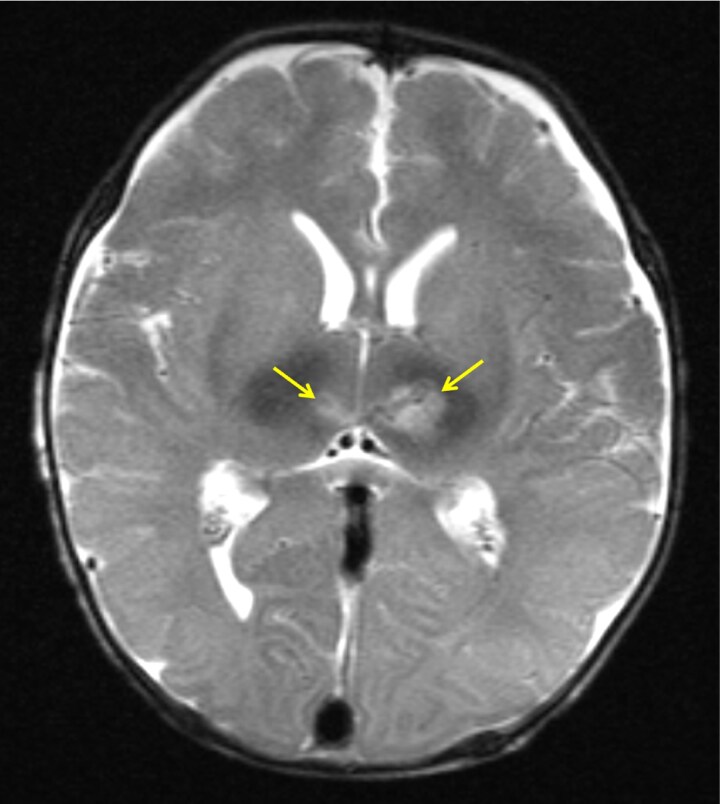
Axial T2 magnetic resonance imaging from hospital day 2 demonstrating bilateral thalamic hyperintensities (arrows).

Abnormal movements were noted on exam, hence a spot electroencephalogram was obtained that revealed multifocal epileptiform discharges from both hemispheres with secondary generalization consistent with subclinical status epilepticus. A head ultrasound subsequently displayed concerning echogenicity within the left thalamus. The day following presentation, magnetic resonance imaging (MRI) of the brain revealed bilateral thalamic T2 hyperintensities and restricted diffusion, left greater than right, potentially consistent with hemorrhage (Fig. 1). A prominent straight sinus was noted, though no vascular occlusion was identified.

## Treatment

The patient arrived to the pediatric intensive care unit in shock with acute hypoxic respiratory failure. His vital signs were significant for hypothermia to 35.5 degrees Celsius and systolic blood pressures in the 40 mm Hg range. He received supportive care that included sedation, intubation and ventilation, IV fluids, and vasopressor support. Management proceeded with bicarbonate infusion and an insulin drip. Pending identification of a potential infectious etiology, he was covered with broad-spectrum antibiotics. Given a suspicion for a mitochondrial disorder, the patient was also started on thiamine, riboflavin, and a multivitamin to bypass various disorders of cellular respiration. With the neurologic features described, the patient was started on multiple antiepileptic medications.

The acidosis resolved rapidly with the described interventions within approximately 12 hours, while other metabolic abnormalities persisted for several days. The patient concurrently began to stabilize. He was weaned off sedatives and pressors and extubated, and parenteral nutrition was transitioned to gavage and then oral feeds. The patient was weaned off his insulin drip on hospital day 22, with blood sugars subsequently in the 150 to 200 mg/dL (SI: 8.3-11.1 mmol/L) range without exogenous insulin prior to discharge. Likewise, coagulation factors quickly normalized on admission, with protein C and antithrombin III activities later obtained; both were within normal, measuring 38.0% (reference range, 24.0-51.0%) and 65.0% (reference range, 60.0-89.0%), respectively. Of note, on hospital day 6, magnetic resonance venography and spectroscopy were obtained and showed no evidence of a thrombus and no lactate peak.

## Outcome and Follow-up

Whole-exome sequencing returned with results indicative of UPD6pat, consistent with the clinical diagnosis of TNDM. Given stable blood glucose levels, the patient was discharged home only with recommendations for monitoring but no exogenous insulin. Glucose levels have remained stable, and the patient remains off insulin to date. Follow-up MRI imaging at 1 year of age demonstrated a small focal area of encephalomalacia and surrounding hemosiderin within the left thalamus consistent with prior hemorrhage. The imaging findings have been accompanied by motor deficits that have improved with physical and occupational therapies but have remained persistent.

## Discussion

Our case report of TNDM with several unusual features extends the spectrum of presentations in the literature. First, the patient described here had multiple blood glucose measurements in the first day of life that were not indicative of hyperglycemia. By our review, the sensitivity of hyperglycemia in the immediate postnatal period for NDM has not been described, nor have factors associated with the timing of presentation of hyperglycemia. Docherty and colleagues shared clinical features of an international cohort of 155 patients with 6q24 TNDM [10]. The mean age at presentation was 8 days with a mode of 1 day, where frequent identification on the first day of life likely reflects surveillance screening in the nursery or neonatal intensive care unit setting. We postulate that the escaped detection in our patient was confounded by the physiology of transitional neonatal hypoglycemia [11], particularly in an SGA baby where glucose stores are limited. The birth parameters of SGA itself support sustained and persistent intrauterine insulin deficiency, where insulin acts as a major fetal growth factor [12], such that postnatal insulin deficiency in the absence of overt hyperglycemia in the first 24 hours seems likely.

At a routine follow-up of the patient at 2 weeks of life, no blood glucose measurements were available; however, there was curiously no indication of poor weight gain that one would anticipate with hyperglycemia and caloric loss through glucosuria. Just 5 days later, laboratory studies revealed a glucose of 1260 mg/dL (SI: 70 mmol/L) with severe acidosis (venous blood gas pH 6.92). A review of data of 88 cases of neonatal diabetes collected from the University of Chicago Monogenic Diabetes Registry highlighted a high frequency of DKA at presentation of 66% [13]. The authors speculated that nonspecific symptoms in noncommunicative infants contributed to the delay in the diagnosis of diabetes and hence progression to DKA. Upon detailed review, only 10 of the 88 patients had 6q24-related NDM, and none of these 10 presented in DKA. Indeed, the absence of DKA at presentation has been promoted as a clinical feature raising suspicion for TNDM, particularly TNDM due to aberrations at the 6q24 locus [9].

With the benefit of hindsight of the natural history and genetic diagnosis, we interpret that all clinical features of the presentation originated from the underlying TNDM, where the limited protective compensatory mechanisms of an SGA infant contributed to rapid clinical deterioration. As the presentation was evolving with features of acidosis, multiorgan failure, negative infectious workup, and bilateral thalamic lesions, there was much discussion for a mitochondrial disorder as the unifying underlying mechanism. Multiorgan involvement is a hallmark of mitochondrial disease, where endocrine dysfunction and diabetes mellitus have been well described, though neonatal presentations with hyperglycemia are rare [14-18]. Moreover, thalamic findings on neuroimaging are found in several mitochondrial disorders, including Leigh syndrome, where focal, bilateral, and symmetric necrotizing lesions of the basal ganglia, thalamus, and brainstem appear as T2 hyperintensities on MRI [19]. A number of other mitochondrial diseases can also involve the thalami, including mitochondrial myopathy, encephalopathy, lactic acidosis, and stroke-like episodes, Kearns-Sayre syndrome, and Alpers syndrome [20]. In our case, we conclude that the thalamic lesions observed represent hemorrhage, where cerebral sinovenous thrombosis that can develop with dehydration has been described in term neonates and can progress to thalamic hemorrhage [21-23]. Thus, the multiorgan failure and thalamic lesions are now understood as a consequence of hyperglycemia, dehydration, ketoacidosis, and a hypercoagulable state and appeared to be a red herring for consideration of mitochondrial disease as the etiology.

## Learning Points

- This study extends the described spectrum of presentation of TNDM to include euglycemia during the first 2 days of life with progression to DKA by 19 days.
- Severe DKA can progress to multisystem organ failure in infancy without other underlying metabolic disease.

## Contributors

All authors made individual contributions to authorship. A.T. and D.J.C. were involved in the diagnosis and management of this patient and manuscript submission. B.M.S. had primary involvement in manuscript preparation and case review. All authors reviewed and approved the final draft.

## Data Availability

Data sharing is not applicable to this article as no datasets were generated or analyzed during the current study.
